# Preliminary assessment of group composition and activity pattern of the critically endangered Bornean Banded Langur *Presbytischrysomelaschrysomelas* in Tanjung Datu National Park

**DOI:** 10.3897/BDJ.12.e124196

**Published:** 2024-06-24

**Authors:** Tukiman Nur-Aizatul, Abd Rahman Mohd-Ridwan, Mohammad Noor-Faezah, Roberta Chaya Tawie Tingga, Mohamad Fhaizal Bukhori, Jayasilan Mohd-Azlan, Azroie Denel, Muhammad Abu Bakar Abdul-Latiff, Badrul Munir Md-Zain

**Affiliations:** 1 Animal Resource Science and Management, Faculty of Resource Science and Technology, Universiti Malaysia Sarawak, 94300, Kota Samarahan, Malaysia Animal Resource Science and Management, Faculty of Resource Science and Technology, Universiti Malaysia Sarawak, 94300 Kota Samarahan Malaysia; 2 Centre for Pre-University Studies, Universiti Malaysia Sarawak, 94300, Kota Samarahan, Malaysia Centre for Pre-University Studies, Universiti Malaysia Sarawak, 94300 Kota Samarahan Malaysia; 3 Department of Biological Sciences and Biotechnology, Faculty of Science and Technology, Universiti Kebangsaan Malaysia, Bangi, Selangor, Malaysia Department of Biological Sciences and Biotechnology, Faculty of Science and Technology, Universiti Kebangsaan Malaysia Bangi, Selangor Malaysia; 4 Institute of Biodiversity and Environmental Conservation, Universiti Malaysia Sarawak, 94300, Kota Samarahan, Malaysia Institute of Biodiversity and Environmental Conservation, Universiti Malaysia Sarawak, 94300 Kota Samarahan Malaysia; 5 Sarawak Forestry Corporation, Kota Sentosa, Sarawak, Malaysia, Kuching, Malaysia Sarawak Forestry Corporation, Kota Sentosa, Sarawak, Malaysia Kuching Malaysia; 6 Environmental Management and Conservation Research Unit (eNCORe), Faculty of Applied Sciences and Technology (FAST), Universiti Tun Hussein Onn Malaysia (Pagoh Campus), 84000, Johor, Malaysia, Muar, Malaysia Environmental Management and Conservation Research Unit (eNCORe), Faculty of Applied Sciences and Technology (FAST), Universiti Tun Hussein Onn Malaysia (Pagoh Campus), 84000, Johor, Malaysia Muar Malaysia

**Keywords:** Colobine, threatened species, behaviour, conservation

## Abstract

The Bornean banded langur (*Presbytischrysomelaschrysomelas*) is critically endangered species primarily found in Sarawak, Malaysia. Albeit this species is in peril, the ecology knowledge of this endemic species of Borneo is still scarce. Thus, a rapid survey employing total count and scan sampling method was conducted between July to August 2023 at Tanjung Datu National Park (TDNP), Sarawak to observe the social interaction of species with the environment. The behaviour of langur was recorded by employing scanning sampling method at 10 minutes intervals. This study sought to provide preliminary data on behavioural ecology of the Bornean banded langur within the national park. During the survey, three groups (consisting two to seven individuals) and a solitary male Bornean banded langur were recorded. The langurs were observed in both dipterocarp forests and coastal forests within the park. The daily activities of the Bornean banded langurs in TDNP were predominantly resting (31%), moving (29%), feeding (26%), vocalizing (14%), but not engaging in other social activities such as grooming, playing and mating. Knowing the behavioural ecological status as well as understanding ecology by identifying the activity pattern of langur is essential to government authorities and pertinent stakeholders to implement conservation strategies for the Bornean banded langur and their habitats.

## Introduction

Tanjung Datu National Park (TDNP), located in Sarawak, Malaysia, is one of the smallest national parks in the region, covering an area of 1379 hectares. Despite its size, it serves as a prime habitat to five diurnal primate species namely long-tailed macaque (*Macacafascicularis* Raffles, 1821), pig-tailed macaque (*Macacanemestrina* Linnaeus, 1766), silver leaf monkey (*Trachypithecuscristatus* Raffles, 1821), Abbott gibbon (*Hylobatesabbotti* Kloss, 1929) and the most threatened species, the Bornean banded langur (*Presbytischrysomelas* Müller, 1838) (Ampeng 2003). The Bornean banded langur is an understudied rare primate and endemic to Borneo. Locally, it is known as penatat or penyatat by Bidayuh, lutung by Malays, luntung in Sarawak Malay, bijit by Iban, and berangad by Kenyah (Mohd-Hatta 2013). Two subspecies have been recognised, namely, *P.c.chrysomelas* and *P.c.cruciger* (Phillipps 2018). The species is distributed from western Sarawak to west Kalimantan, Indonesia (Phillipps 2018). Historically, this species was widely dispersed throughout Borneo.

Nevertheless, in recent years, *P.c.chrysomelas* is restricted in its range to five specific areas in Sarawak, namely Tanjung Datu National Park (TDNP), Samunsam Wildlife Sanctuary (SWS), Maludam National Park (MNP), Similajau National Park (SNP), and Pueh National Park (PNP) (Noor-Faezah et al. 2023). The native habitat of langur has been constricted due land conversion for agriculture and urban development purposes, exacerbating its vulnerability. Currently, the species occupies less than 5% of their lowland habitats, namely tropical rainforests, mangrove forests, and swamp forests (Nijman et al. 2020).

Bornean banded langur groups are often found in three to seven individuals, with one male, multi females and their offsprings. The species is known to eat 25% to 65% of fruits and leaves (Ehlers Smith 2014, Nijman et al. 2020). According to the International Union for Conservation of Nature (IUCN) (2022), the primate is one of the world's 25 most endangered primate species and it is on the brink of extinction (Mittermeier et al. 2022). *Presbytischrysomelaschrysomelas* has been deemed as Critically Endangered in the IUCN Red List (Nijman et al. 2020). Yet, scientist have paid little attention to it. Thus, ecological information on this species remains limited compared to other iconic Bornean species such as proboscis monkeys and orangutans.

In comparison with other *Presbytis* species, the information on the ecology of *P.c.chrysomelas* is limited and inconclusive with the research on ranging behaviour conducted by Ampeng and Md-Zain (2012). They discovered the ranging patterns employed by 17 groups of *P.c.chrysomelas* in SWS encompass vertical, cross-horizontal, and straight horizontal movements. The Bornean banded langur displays a short daily movement distance which might be influenced by the abundance and distribution of food resources. Conversely, study on sister subspecies, Tricolor langur (*P.c.cruciger*) in Danau Sentarum National Park (DSNP) comprehend the key ecological characteristics including their population size, habitat characteristics, feeding ecology and behavioural ecology (Musyaffa and Santoso 2020, Santoso et al. 2023). They found that Tricolor langur inhabits both primary and mixed forest. The population of diurnal primates in TDNP including *P.c.chrysomelas* (previously known as *P.melalophos*) was first conducted two decades ago by Ampeng (2003). The study however did not provide the detailed information on the population size and group composition of the species.

Despite the reported decline in the population of *P.c.chrysomelas* (Nijman et al. 2020), the status of this species in TDNP is still relies on data from 20 years ago (Ampeng 2003). Hence, extensive, and conclusive behavioural ecology study is timely to provide updated data, notably on its population size and social behaviour. This data is crucial for assessing the current conservation status of *P.c.chrysomelas* 20 years after the first study. It also plays important role in assisting policymakers or authorities in developing and establishing action plans to sustain the remaining wild population *P.c.chrysomelas* from extinction. Thus, this study aimed to provide updated information on population size, group composition and behaviour of Bornean banded langur, *P.c.chrysomelas* in prime habitat area in Tanjung Datu National Park, Sarawak.

## Material and methods

### Study Site

**Figure 1.**
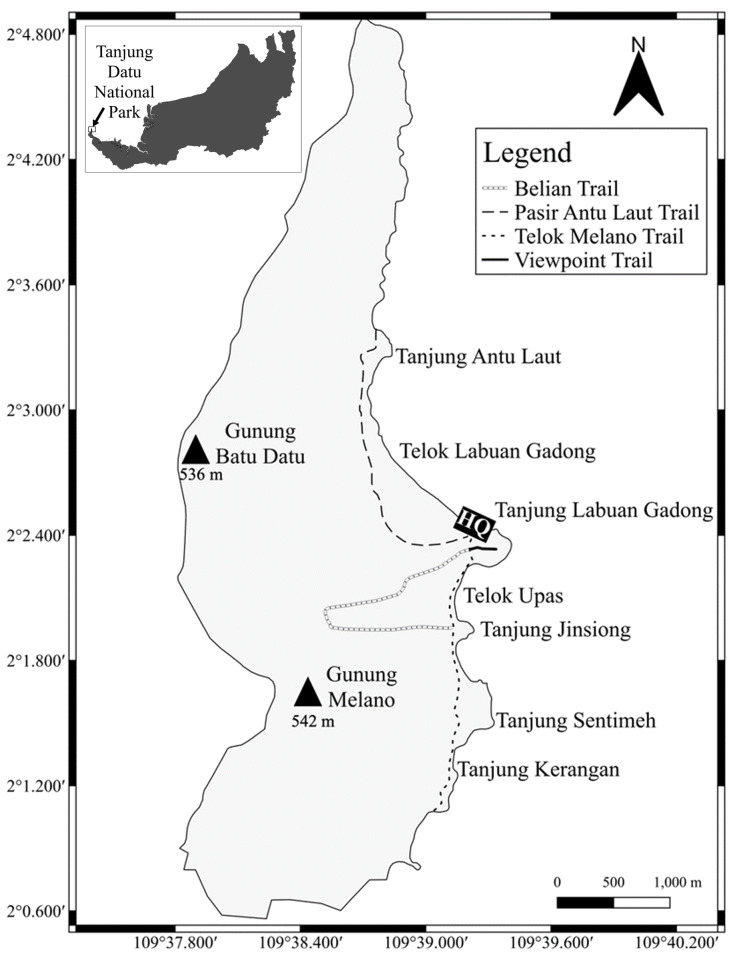
The map of Tanjung Datu National Park.

This study was conducted at Tanjung Datu National Park (TDNP), Lundu, Sarawak, Malaysia (2°2'32.80"N 109°38'50.71"E). This site is located on the western tip of Sarawak’s coastline (Fig. 1). The park was declared as National Park in 1994 and spans 1,379 hectares of five habitat types. TDNP was dominated by mixed dipterocarp forest and covers the flank of the mountains (Hazebroek and Abang Morshidi 2000). Other vegetation found in this park includes kerangas forest, the mangrove forest, the coastal forest, the sub-montane forest, and the secondary forest (Mohd-Azlan et al. 2018). The main topography is a slender crest of steep hills reaching an elevation of 543 meters above sea level, with its highest peak named Gunung Melano. Beyond the park lie unspoiled white-sand beaches, clean waterways, and regions teeming with coral reefs, all of which are surrounded by verdant rain forests (Abdul Rahman et al. 2015).

### Field Observation

Four surveys were conducted at TDNP between July and August 2023 to record the population size and behaviour activities of Bornean banded langur. Surveys were done around the park’s headquarters and three main trails namely, Belian Trail, Telok Melano Trail and Pasir Antu Laut Trail. Data collection, including population census and behaviour surveys were conducted twice per day; 6.30 am to 10.00 a.m. and 12.00 noon to 6.30 p.m. These periods were chosen based on previous reports of the active and foraging time for the silvered langur (Laman et al. 2007, Wan Azman 2017) and the Bornean banded langur (Ampeng and Md-Zain 2007). The group composition of the Bornean banded langur was investigate using total count method (Sutherland 2006). Following Najmuddin et al. (2021), the counted individuals were classified based on their body size and age-sex characteristics (adult male, adult female, sub-adult, juvenile, and infant), with sub-adults appear smaller than adults. Meanwhile, activity pattern of the Bornean banded langur were observed using scan sampling method within 10 minutes intervals and 5 minutes rest from left to right or clockwise direction to avoid repeated sampling (Altmann 1974). Data for activity patterns were recorded and classified as moving, resting, feeding, vocalizing, and others activity categories (Najmuddin et al. 2020) as described in Table 1. Each activity was calculated as percentage of its frequency.

## Results

### Group composition

**Figure 2.**
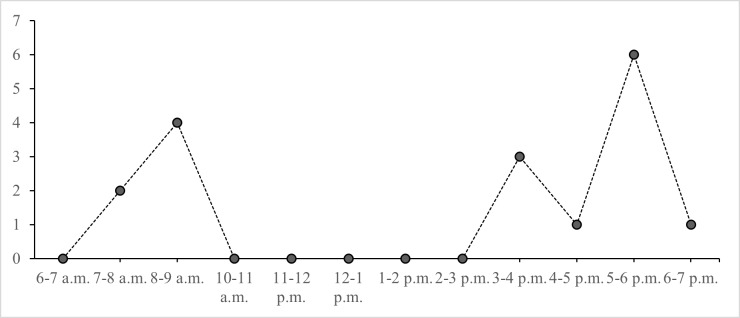
Activity pattern of *P.c.chrysomelas* by hour.

Overall, three groups and one solitary of Bornean banded langur were recorded with a total of 17 encounters during the survey (Fig. 2). Each group consisted of one adult male and groups of two to seven individuals (Table 2). The first group recorded was a group of seven individuals, which consisted of an adult male, adult and sub-adult females and an infant. Meanwhile, other groups consist of two and three individuals, respectively. These langur groups were often encountered in the mixed-dipterocarp forest, mainly in Pasir Antu Laut Trail. A solitary male was also recorded in this study, usually sighted near the Park Headquarters (2°2'32.80"N 109°38'50.71"E) and the beach forest.

### Activity pattern

**Figure 3.**
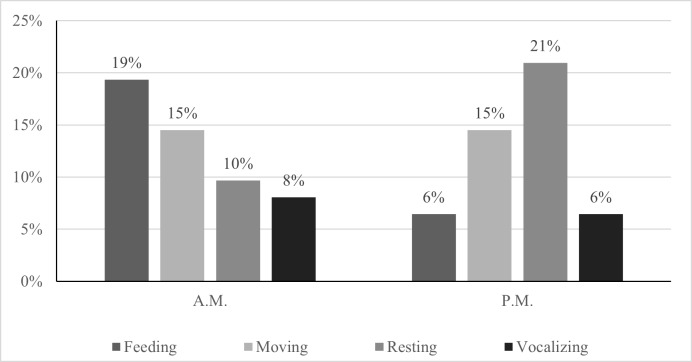
Daily activity pattern of *P.c.chrysomelas* in TDNP at different time.

The study spanned a total of 147 sampling hours. Three peaks of langur activities were observed in mid-morning 8 a.m. to 9 a.m., afternoon between 3 p.m. to 4 p.m. and early evening between 5 p.m. to 6 p.m. (Fig. 2). Langur started to be active at 6 a.m. Langur allocated a significant portion of their morning in feeding (19%) but comparatively lower in the afternoon (6%). In contrast, resting was less frequent during the earliest of the day (10%), however it reached its peak during the latter hours of the day (21%). Langur were seen moving almost all the time. They spent 15% of their time moving, both in the morning and afternoon. Vocalization was more frequent in the morning (8%) compared to the latest hours of the day (6%) (Fig. 3). Overall, langurs spent 31% of their time resting, 29% moving, 26% feeding and 14% vocalizing. Meanwhile, other social activities such as grooming, playing, and mating, as well as agonistic were not recorded in this study.

## Discussion

### Group composition

The composition of langur groups in TDNP varies, with group sizes ranging from one to seven individuals. Notably, this range roughly corresponds to the general trend reported among Bornean banded langurs, where group sizes typically vary from three to seven individuals per group (Nijman et al. 2020). The present population of Bornean banded langur in TDNP (n=13) is lower than the population 20 years ago (n=25) as reported by Ampeng (2003). Additionally, Ampeng and Md-Zain (2012) findings show that the Bornean banded langur population in TDNP is smaller than the population in SWS, where groups vary from 8 to 13 individuals. Meanwhile, the population size of the Tricolor langur (*P.c.cruciger*) in DSNP ranged from 20 to 24 individuals (Santoso et al. 2023).

#### Activity Pattern

**Figure 4.**
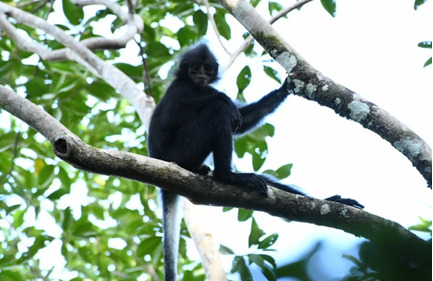
*Presbytischrysomelaschrysomelas* perched on a tree branch.

The activity pattern of the Bornean banded langur indicates that a significant portion of their daily activities is allocated to resting. This finding aligns with similar observations made in other colobine species, including Banded langur (*P.femoralis*) (Najmuddin et al. 2021), red langur (*P.rubicunda*) (Ehlers Smith et al. 2013), Mentawai langur (*P.potenziani*) (Hadi 2011), proboscis monkey (*N.larvatus*) (Matsuda et al. 2009), and silvered langur (*T.cristatus*) (Akbar et al. 2021). The Bornean banded langur were often seen resting in position where they sat in a hunched position (Fig. 4) with one hand grasping the nearest branch of the tree. Langur rest more during the afternoon due to several factors such as postprandial metabolic processes, or the influence of the surrounding temperature on body condition (Zhou et al. 2022).

Comparatively, the Bornean banded langur spend more time feeding (26%) than other closely related species such as *P.femoralis*, which allocate 21% of their time to feeding (Abdul-Latiff et al. 2019), 12.1% for *T.cristatus* (Akbar et al. 2021) and 12.6 % for *T.obscurus* (Siti-Kauthar et al. 2019). The diet of langur in this survey consisted mostly of leaves (81%) such as Resak Laru (*Vaticapauciflora)* and Mengkudu Besar (*Morindacitrifolia*) and 19% of fruits, notably Kerueh (*Lophopetalumpallidum*). Consistent with previous studies (Fleagle 1988, Eliana et al. 2017, Barbhuiya et al. 2022), folivores were found to spend more time resting and less time feeding compared to frugivores and insectivores. Additionally, folivores tended to exhibit shorter day ranges and smaller home ranges, particularly in forested habitats where leaves are abundant and evenly distributed (Dasilva 1992, Ehlers Smith 2014, Bach et al. 2017). This information sheds light on the notably high resting activities observed in this study, emphasizing the influence of dietary habits and environmental factors on the behavioural patterns of the species.

The percentage of moving activity was comparable both in the morning and evening, as langurs persistently engaged in movement until they have selected the ideal feeding trees required by the group (Santoso et al. 2023). This primate is an arboreal animal in which feeding, resting, moving and social activities were predominantly at forest canopy. However, they were once sighted on the ground before climbing on tree upon seeing researcher as they were not habituated to human presence. Similarly, study in DSNP found that *P.c.cruciger* descend to pick up the fallen food (Santoso et al. 2023). Langur in their natural habitat rarely exhibits terrestrial behaviour. However, such behaviour was frequently seen in *P.rubicunda* at disturbed habitat (Cheyne et al. 2018). *Presbytischrysomelaschrysomelas* usually found foraging and resting in beach forest in this study. An earlier study by Mohd-Azlan et al. (2018)reported eight instances of *P.c.chrysomelas* on ground in the beach forest of TDNP.

Vocalization took up 14% of the day in this study compared to only 1.73% and 4% in *P.femoralis* (Abdul-Latiff et al. 2019, Najmuddin et al. 2020), 0.8% in *T.obscurus* (Siti-Kauthar et al. 2019) and 3.89% in *T.obscurus* (Md-Zain and Ch'ng 2011). *Presbytischrysomelaschrysomelas* often initiates vocalizations in the early morning before leaving their sleeping tree, to communicate with other group members and coordinate foraging activities. During the observation, langur often vocalizes while moving. Vocalization is essential in primates to promote cohesion among group members (Riley 2005), and also to warn other group members when they are in threat or to attract potential predators such as hawk eagles, a known predator to langurs (Nijman and Nekaris 2012, Hankinson et al. 2022, Santoso et al. 2023).

Compared to other studies (Matsuda et al. 2009, Ehlers Smith et al. 2013, Abdul-Latiff et al. 2019, Siti-Kauthar et al. 2019, Najmuddin et al. 2020, Akbar et al. 2021, Santoso et al. 2023) social activities such as grooming, mating, playing, and agonistic were not recorded in this study. It might be due to several factors, including the dense forest canopy at the study site, which limit observers vision and make it difficult to observe primate behaviours. Besides, the langurs were not habituated to human presence, causing them to flee upon seeing researchers. Also, this study was conducted in a short period (2 months), compared to other studies that were conducted for few months or throughout the year (Siti-Kauthar et al. 2019, Najmuddin et al. 2020, Akbar et al. 2021, Santoso et al. 2023).

### Conservation Significance

Data on primate ecology, particularly *P.c.chrysomelas* in TDNP is objectively essential for the park management unit to provide information on interspecific interaction among arboreal primates and engage in habitat conservation activities. These activities may include planting food plant species for enrichment and implementing visitor control measures. Obtaining data on *P.c.chrysomelas* population via monitoring the population growth and trend is needed to ensure the long-term survival of *P.c.chrysomelas* in TDNP. Study on behavioural ecology, notably activity budget analysis, is also of utmost importance for comprehending how primates utilise their environments, allocate resources, and respond to environmental changes (Naher et al. 2022, Kshetri et al. 2023). Moreover, the data also facilitate the implementation of habitat enrichment, security measures, and additional study pertaining to the ecology and conservation of langurs and its habitat by the park management unit and other relevant stakeholders.

## Conclusions

The current population of the Bornean banded langur in TDNP is lower than the population recorded 20 years ago. Albeit an alarmingly low number, it is positive to note its existence in the park. This data establishes a baseline information for the observed group of *P.c.chrysomelas* in the park. The activity pattern of the species was divided into two parts; morning (A.M.) that was dominated by feeding activity (19%) and afternoon (P.M.) dominated by resting activity. Generally, langur allocated most of their time in resting, followed by moving, feeding and vocalization consistent with previous studies. The Bornean banded langur were found in both mixed-dipterocarp forest and beach forest. Their diet preference mostly consisted of leaves and fruits such as Resak Laru (*Vaticapauciflora*), Mengkudu Besar (*Morindacitrifolia*) and Kerueh (*Lophopetalumpallidum*). Knowing the population status as well as understanding ecology by identifying the activity pattern, feeding and habitat preferences of langur is essential to the government authorities and pertinent stakeholders to effectively execute conservation plans for the Bornean banded langur and its habitat. Furthermore, the present data serve as evident of importance of conserving TDNP as the prime habitat ensure survival of the critically endangered *P.c.chrysomelas* in Sarawak.

## Figures and Tables

**Table 1.** Description of each behaviour categories.

**Behaviour**	**Description**
Moving	Walking, jumping, leaping or climbing
Resting	Inactive, sleeping, sitting, or lying
Feeding	Foraging, manipulating, and ingesting the food materials
Vocalization	Emit sound, or long calling.
Others	Grooming, playing, mating, and aggression

**Table 2.** Group size and composition of *P.c.chrysomelas* in TDNP.

**Category**	**Group 1**	**Group 2**	**Group 3**	**Solitary**	**Percent Composition**
Adult Male	1	1	1	1	31%
Adult Female	3	1	1		38%
Sub-adult	2				15%
Juvenile		1			8%
Infant	1				8%
**Total**	**7**	**3**	**2**	**1**	**13 (100%)**
